# Ameliorative Effect of *Artemisia absinthium* Ethanolic Extract Against Sodium Fluoride Toxicity in Rat Testes: Histological and Physiological Study

**DOI:** 10.3390/vetsci12040371

**Published:** 2025-04-15

**Authors:** Sawsan A. Ali, Zainab A. H. AL-Mousawi, Ahlam A. AL-Rikaby, Sameh Mohamed Farouk, Shaaban S. Elnesr

**Affiliations:** 1 Department of Anatomy and Histology, College of Veterinary Medicine, University of Basrah, Basrah 61004, Iraq; sawsan.ali@uobasrah.edu.iq; 2 Department of Physiology and Pharmacology, College of Veterinary Medicine, University of Basrah, Basrah 61004, Iraq; zainab.hassan@uobasrah.edu.iq (Z.A.H.A.-M.); ahlam.abdulnabi@uobasrah.edu.iq (A.A.A.-R.); 3 Cytology and Histology Department, Faculty of Veterinary Medicine, Suez Canal University, Ismailia 41522, Egypt; dr_smf_hist@vet.suez.edu.eg; 4 Department of Poultry Production, Faculty of Agriculture, Fayoum University, Fayoum 63514, Egypt

**Keywords:** fluoride, *Artemisia absinthium*, rat testis, androgen receptor, antioxidant

## Abstract

This study aimed to investigate the effect of *Artemisia absinthium* extract on testicular dysfunction in rats and explain the involvement of the androgen receptor signaling pathway as a biomarker in maintaining fertility during sodium fluoride (NaF) treatment. Thirty-two male rats were divided equally into four groups and received treatment for 60 days. The control group (I) received normal saline; group II received Artemisia extract at 100 mg/kg b.w.; group III received NaF at 12 mg/kg b.w. orally; and group IV received NaF (12 mg/kg b.w.) and Artemisia extract (100 mg/kg b.w.). The testis weights and the lipid peroxidation, luteinizing hormone (LH), follicle-stimulating hormone (FSH), and testosterone levels were estimated. The genital organs were prepared and immunoreacted with a receptor. Histomorphometric analyses were performed to obtain the diameter of the seminiferous tubules and the height of the germinal epithelia in the testes. The results showed that exposure to NaF caused a significant increase in testis weight and malondialdehyde (MDA) and a decrease in serum LH, FSH, and testosterone concentrations compared to the control group, while extract administration induced an increase in the levels of these hormones in group IV. Testicular histological and histomorphometric changes were observed in group III: degenerative seminiferous tubules with vascular congestion, disorganization of the germinal layer, and decreased seminiferous tubule diameter and germinal epithelium height. The expression of androgen receptors in the testes of the NaF-treated rats was significantly reduced. In contrast, these testicular histological changes were ameliorated in rats treated with the extract. The results allow us to conclude that the administration of *Artemisia absinthium* confers positive effects on male reproductive function by inhibiting fluoride, maybe via ameliorative testicular function.

## Simple Summary

This study examined the ameliorative effect of an ethanolic extract of *Artemisia absinthium* against sodium fluoride toxicity in rat testes through histological and physiological testing. The results showed that the administration of *Artemisia absinthium* confers positive effects on male reproductive function by inhibiting fluoride, maybe via ameliorative testicular function.

## 1. Introduction

Fluorides are fluorine-containing organic and inorganic substances. Fluorine can combine with any element except helium or neon to generate a wide range of fluorine-containing compounds [1,2]. The World Health Organization recommends a fluoride level of 0.5 to 0.8 mg/L in drinking water, with a maximum of 1.5 ppm [3]. The biochemistry and physiology of fluoride in the human body disprove the belief that fluoride is inert and, thus, harmless in the human body [4].

Most fluoride enters the body through the gastrointestinal tract, where it is absorbed easily in the stomach, given the absence of a specific enzyme system [5]. Fluoride is quickly deposited in the skeleton or eliminated through the kidneys once it reaches the plasma. The absorption of skeletal fluoride is influenced by a variety of factors, including bone modeling and remodeling activity, as well as age [6].

Fluoride is also secreted in saliva. The fluoride that is not deposited in bone is mostly eliminated through the kidneys, with only a small amount passing through the stool. The renal clearance of fluoride from plasma is regulated by both urine flow and pH [5]. Nausea, vomiting, and a reduction in blood calcium are common indications and symptoms of acute fluoride toxicity, which can cause local or systemic signs of muscle tetany. Coma, convulsions, and cardiac arrhythmias are additional symptoms, as are stomach cramping and pain, as well as rising hypocalcemia and hyperkalemia. Excess fluoride consumption usually results in death within four hours; the prognosis is good if the person survives for 24 h [7].

Several in vitro and in vivo studies have found that NaF negatively affects the operation of the female and male reproductive systems [8,9,10]. Rat studies have shown that fluoride exposure results in oxidative stress, reduced sperm motility and density, and histological changes in testicular tissue, which can lead to infertility. Sodium fluoride has also been connected to decreased levels of sex hormones, such as testosterone, which is crucial for the health of the male reproductive system [11].

Throughout Asia, the Middle East, North Africa, and Europe, *Artemisia absinthium* L. (*A. absinthium*) has been utilized as an herbal medicine. *A. absinthium*, also called wormwood, is a highly therapeutic plant that is nearly universally listed in herbal medicine manuals. Wormwood is a member of the Asteraceae family, the Artemisia genus, and the Plantae kingdom [12]. *Artemisia absinthium* L. is rich in nutrients and phytochemicals, including total phenolic acids like syringic acid, quercitin-3-o-rhamnoglucoside, quercetin, rutin, and other flavonoids and glycosides such as essential oil, isoquercitrin, quercitin-3-o-d-glucoside, anabsinthin, anabsin, artabsin, absinthin, organic acids, matricin, resins, lactones, and absin [13].

These pharmacophores have substantial antioxidant, anti-inflammatory, and free radical scavenging properties [14]. *A. absinthium* is used as an antimalaria and anticancer agent and as an insecticide, diuretic, vermifuge, and antispasmodic; it has been shown to be useful in the treatment of cough, diarrhea, and the common cold [15]. The aims of this study included examining the ameliorative effect of an ethanolic extract of *A. absinthium* against tissue damage and oxidative stress induced by fluoride in rat testes.

## 2. Materials and Methods

### 2.1. Chemicals

Sodium fluoride (NaF) was administered three times per week at a dose of 12 mg/kg b.w. This dose was chosen in accordance with Chen et al. [16].

### 2.2. Preparation of Artemisia absinthium Extract

An *Artemisia absinthium* L. plant was obtained at a local market in Basrah, Iraq. The plant was dried in the shade and then ground; 60 gm of powder was added to a bottle flask in a chemical mixer along with 500 mL of 70% ethanol and was then extracted for 24 h at 50 °C. Whatman filter paper was used to filter the extract, and then an evaporation process was applied to remove the solvent from the extract using a rotary evaporator. Finally, the dry extract was collected and stored at 4 °C until use [17].

### 2.3. Experimental Animals

Thirty-two healthy male adult albino rats (Wister strain) weighing 180–230 g (7–8 weeks old) were obtained for this study. The animals were housed in appropriate cages under standard management conditions of 20–25 °C, 5% humidity, and a light period of 12 h. They were given a standard diet along with ad libitum water. This study was conducted in the animal house at the College of Veterinary Medicine, University of Basrah, Iraq, with IACUC ethical permission for animals (26/37/2024).

### 2.4. Design of Experiments

Following acclimation, the rats were separated into four groups, with eight animals in each:

Group I: normal male rats receiving normal saline (negative control);

Group II: male rats treated with ethanolic extract of *A. absinthium* (100 mg/kg b.w. orally) for 60 days;

Group III: a positive control group of male rats receiving NaF (12 mg/kg b.w. orally) three times weekly for 60 days;

Group IV: male rats receiving NaF (12 mg/kg b.w. orally) three times weekly and ethanolic extract of *A. absinthium* (100 mg/kg b.w. orally) 1 h later for 60 days.

The body weights of the animals and the weights of the testes were measured using a digital balance. At the end of the study, cardiac blood samples were collected from all the rats after anesthetization; the blood samples were poured into plan tubes and centrifuged to obtain the serum, which was stored at −20 °C for hormone and malonedialdehyde (MDA) assays.

### 2.5. Blood Assays

Fortress’s kit for measuring testosterone and Monobind Inc.’s kit (Lake Forest, CA, USA) for measuring follicle-stimulating hormone (FSH) and luteinizing hormone (LH) were used in the enzyme-linked immunosorbent assay (ELISA) method for measuring hormones. In accordance with Buege and Aust, for MDA serum estimation, thiobarbituric acid (TBA) reactivity was used to evaluate serum lipid peroxide levels [18].

### 2.6. Histological Examination

The testes were removed from the rats and directly fixed in Bouin’s solution for 12 h; then, 70% alcohol was used to rinse the Bouin’s fixative off the samples. The tissues were dehydrated in increasing concentrations of ethanol before being treated with xylene and embedded in paraffin. Hematoxylin and eosin staining was used to stain five-micron-thick slices of paraffin-embedded tissues placed on glass slides. A light microscope was used to analyze slides prepared from the middle portions of the testis tissue for compositional and functional alterations [19].

### 2.7. Immunohistochemical Staining of Androgen-Receptor Protein

First, testicular sections were treated with 3% H_2_O_2_ for 10 min, followed by washing of the sections with phosphate buffered saline (PBS, 0.01 M, pH 7.4) for 5 min each. The sections were then incubated with an anti-mouse primary antibody (androgen receptor-antibody) in a citrate buffer at 37 °C for 2 h. Kits were obtained from Proteintech. After that, slides were washed in PBS three times for 5 min and then treated with biotinlyted secondary antibody and streptavidin-horseradish peroxidase (HRP) enzyme for 20 min in order to observe primary antibody-bound biotin [20]. A power stain (1.0) Sav-HRP DAB kit (Genemed Biotechnologies, South San Francisco, CA, USA), (A-USA) was used to visualize the antigen–antibody reaction in the samples. Then, DAB chromogen was prepared and applied for 5 min. The slide was counterstained with a sufficient amount of Mayer’s hematoxylin [19]. After the background noise was eliminated, the color intensity of the immunoreactive regions was employed as the standard of cellular activity for quantitative measurement.

### 2.8. Imaging and Histomorphometric Analysis

Histomorphometric measurements were performed using a Leica Qwin 500 at the Anatomy and Histology Department, College of Veterinary Medicine, University of Basrah. Rat testis tissue was examined using light microscopy at magnifications of ×100 and ×400, and the images were transferred to a monitor to determine the diameters of the seminiferous tubules and the heights of the germinal epithelial layer. Immunohistochemical analysis was evaluated by using Image J software (version 1.54) according to Mane et al. [21] for quantitative estimation of immunoreaction of protein expression in testicular tissues.

### 2.9. Statistical Analysis

The results of the current study were evaluated using a one-way covariance (ANOVA) test. The statistical program SPSS V. 17 was used to perform all statistical computations (SPSS Inc., Chicago, IL, USA). Means and standard deviations (X SD) were used to represent the data [22].

## 3. Results

### 3.1. Effect of Ethanolic Extract of A. absinthium on Body and Testis Weights Under NaF Toxicity in Rats

The results presented in Table 1 reveal that the body weight in the positive control group decreased significantly (*p* ≤ 0.05) compared to that in the negative control group (I) and the other treatment groups, whereas the body weight in the *A. absinthium* ethanolic extract + NaF group (IV) increased compared to that in the positive control group (II).

There were no significant differences in body weight between the rats given the extract treatment and those in the negative control group (*p* > 0.05). However, there was a significant decrease (*p* ≤ 0.05) in the testis weight of the positive control rats compared to the negative control rats and those in the other treatment groups, while the testes of the rats treated with *A. absinthium* ethanolic extract + NaF showed a significant increase (*p* ≤ 0.05) in weight compared to the testes of rats treated with extract alone (III) and those from the negative control group.

### 3.2. Effects of Ethanolic Extract of A. absinthium on Levels of FSH, LH, Testosterone, and MDA Under NaF Toxicity in Male Rats

Table 2 presents the effect of *A. absinthium* ethanolic extract on serum FSH, LH, and testosterone levels in male rats. The outcomes reveal a substantial drop (*p* ≤ 0.05) in FSH, LH, and testosterone levels in the positive control group compared with the other groups. However, there were non-significant changes (*p* > 0.05) in FSH, LH, and testosterone levels in the serum of rats treated with *A. absinthium* ethanolic extract compared to the negative control group. The results also indicate a significant increase (*p* ≤ 0.05) in FSH, LH, and testosterone in the serum of rats treated with *A. absinthium* ethanolic extract + NaF compared to the positive control group. The serum MDA levels of rats in the positive control group increased significantly (*p* ≤ 0.05) compared to those in the other groups, while the results show a significant decrease (*p* ≤ 0.05) in MDA in the serum of rats treated with *A. absinthium* ethanolic extract compared with the positive control group.

The results in Table 3 reveal significant decreases in the diameter of seminiferous tubules and the height of the germinal epithelium in testes from group III compared to the control groups and group II. The statistical significance of these results was also analyzed. In the table below, the data show that the decreases in the diameter of histological tubules and the height of the germinal epithelium in the testes were significantly different for group III compared to the control groups and group II.

In the NaF- and extract-treated group (IV), there was a significant increase compared with the positive control group (III). Besides this, there were no significant (*p* > 0.05) differences observed between the negative control group (I) and group II. The histological findings induced by NaF in testis tissues were evaluated using hematoxylin and eosin staining.

**Figure 1:**
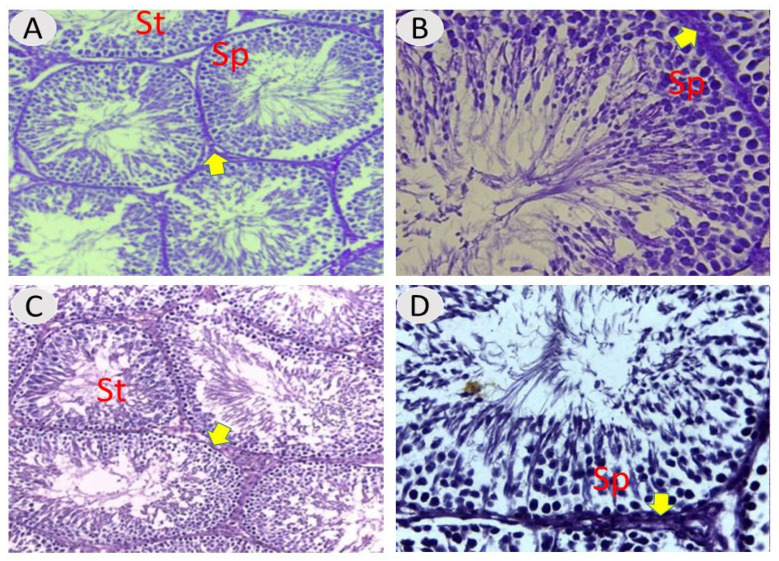
(**A**,**B**) Testicular section from untreated control group showing normal histological architecture of active mature seminiferous tubules (St), complete spermatogenic series (Sp), and interstitial regions (thick arrows). (**C**,**D**) Testis of a rat given *A. absinthium* extract at 100 mg/kg b.w. for 60 days, showing a normal histological structure of most seminiferous tubules. H and E staining, 100× and 400× magnification.

**Figure 2:**
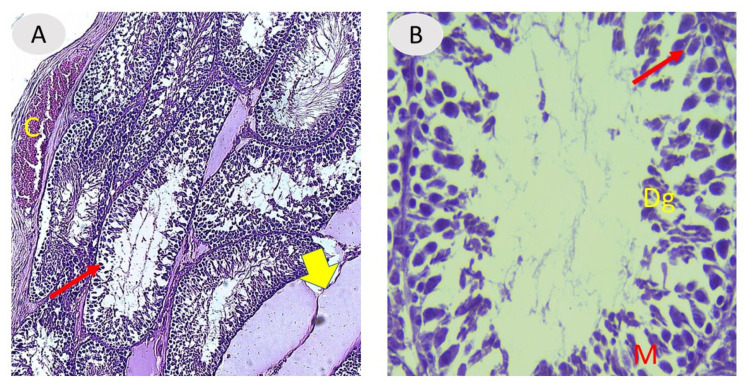
(**A**,**B**) Testicular section from the NaF-treated group (III) showing degeneration of seminiferous tubules (arrows), congestion in blood vessels (C), and degeneration with enlarged interstitial spaces (thick yellow arrow). (**B**) Magnified section of seminiferous tubules from the NaF-treated group (III) shows depletion of germ cells (Dg) with microvaculation (M) and necrosis in germ cells (arrow). H and E staining, 100× and 400× magnification.

Figure 1 shows testes from control rats and rats treated with *A. absinthium*, displaying the typical histological composition of active mature functioning seminiferous tubules surrounded by tunica propria and lined with germinal cells and Sertoli cells associated with complete spermatogenic series. After 60 days, the testes from the NaF group showed marked degeneration in most of the seminiferous tubules, with depletion of the spermatogenic series, congestion in testis blood vessels, degeneration, and enlarged interstitial regions. A magnified section displayed marked thickening in the basement membrane in some tubules and microvacuolation occurring in the basal layer, with necrosis in the germ cells (Figure 2).

**Figure 3:**
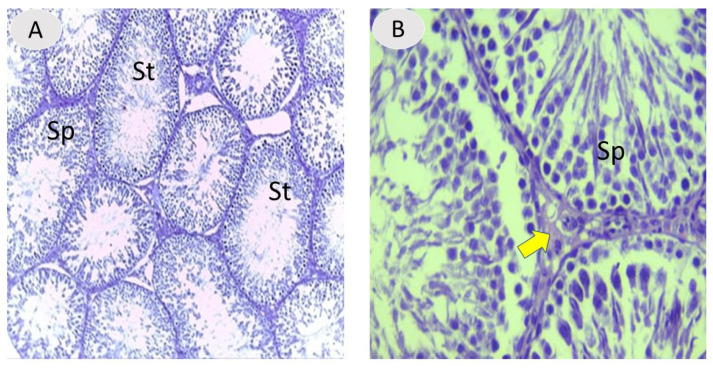
(**A**,**B**) Testicular section from a rat treated with NaF and *A. absinthium* showing improved histological structure of the seminiferous tubules with restoration of spermatogenic series (SP). (**B**) Magnified section of seminiferous tubules (St) from group IV shows further rebuilding of spermatogenic series (SP) with restored interstitial cells (thick yellow arrow) and reduced thickening of basement membrane. H and E staining, 100× and 400× magnification.

**Figure 4:**
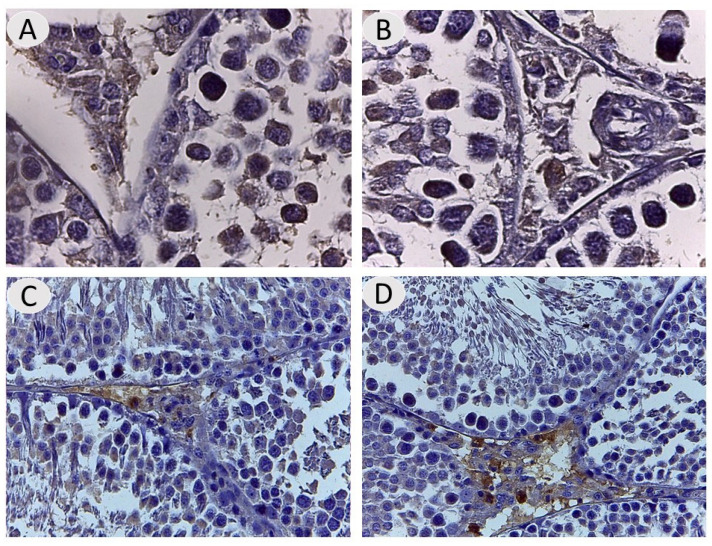
(**A**,**B**) Testes of control rats and those given *A. absinthium* reveal strong immunohistochemical expression of androgen via nuclear staining of interstitial cells. (**C**) Testis of an NaF-treated rat showing lack of immunoexpression of androgen receptor. (**D**) Testis of an NaF- and *A. absinthium* extract-treated rat showing moderate androgen receptor nuclear immunoexpression in interstitial cells.

The testes of the NaF-treated rats given *A. absinthium* extract appeared to ameliorate histological structure and regular arrangement of germinal cells in most seminiferous tubules compared to the positive control, with the restoration of the spermatogenic layer and reduction of thickening basement membrane with more regeneration of the interstitial region (Figure 3), which indicates the effectiveness of the extract in decreasing the destructive effects of fluoride in the testis inducer. With regard to immunohistochemistry of androgen receptor (AR), an assessment of AR distribution was mostly in testicular interstitial areas with high expression and largely observed in the nuclei of the Leydig cells, endothelial cells, and peritubular cells in the interstitium. Also, AR was localized to the nuclear germinal cells in seminiferous tubules. Androgen expression was 33%, 26%, 7%, and 17% in the different experimental groups (A, B, C, and D, respectively). Our results showed that there was a marked reduction in the AR immune reactivity in the testes of the NaF-treated group compared to the other groups (Figure 4).

## 4. Discussion

The results of this study indicate that sodium fluoride affects the structure and function of rat testes. At the end of the experiment, the animals in the fluoride-treated group displayed a significant loss of body weight; this may have been caused by decreased food consumption, which also reduces protein synthesis and energy metabolism [23,24]. However, when rats were treated with an extract of *A. absinthium*, their body weight increased because the extract contains high levels of protein, essential amino acids, minerals, vitamins, antioxidants, and flavonoids [25], which are crucial for growth and weight gain in rats. According to our findings, fluoride may inhibit Sertoli cells’ ability to express androgen receptor mRNA, which reduces the number of androgen receptors available for testosterone to act on. Fluoride also inhibits testicular zinc levels and affects angiotensin-converting enzyme activity, which in turn inhibits spermatogenesis. Additionally, it causes a significant decrease in epidermal growth factor, spermatocytes, and spermatogonia through its receptors in Leydig’s cells, which are crucial for male animal reproduction [26,27]. Fluoride poisoning reduces the activity of some enzymes and thus has a negative impact on metabolic processes like glycolysis, protein synthesis, and antioxidative pathways. These modifications, combined with a decrease in food intake, resulted in a drop in body weight, which, in turn, caused a decrease in testicle weight [27,28]. The weight of this organ may change in response to any change in androgen concentration [26].

The pituitary gland may be impacted by sodium fluoride, which would result in lower levels of gonadotropin-releasing hormone, FSH, and LH, which would then lead to lower levels of steroid biosynthesis [29]. A decrease in testosterone levels in the positive control group was demonstrated in our study through histological changes in the testes, confirming a lack of spermatogenesis. The results of our study provide evidence that oxidative stress brought on by sodium fluoride may contribute to the pathogenesis of testicular dysfunction, as shown by histopathological changes. The present study’s findings revealed a significant increase in the serum concentrations of testosterone, LH, and FSH in rats treated with *A. absinthium* ethanolic extract + NaF compared to the positive control group, possibly due to *A. absinthium*’s antioxidant properties, which can lessen the toxic effects of sodium fluoride on testicular function. Antioxidants reduce the production of reactive oxygen species through a variety of mechanisms, such as scavenging the byproducts of lipid peroxidation during free radical scavenging and blunting oxide and hydrogen peroxide [30].

Furthermore, sodium fluoride significantly increased MDA levels, indicating the induction of oxidative stress [31]. Treatment with *A. absinthium* ethanolic extract affected MDA, an oxidative stress parameter, because the extract contains many compounds like polyphenolics and flavonoids, which are powerful antioxidants and remove free radicals [32]. Besides this, fluoride caused a decrease in the height of the germinal epithelium in the positive control group. Sodium fluoride reduces the metabolic activity of germinal cells, enlarging the interstitial space and consequently causing tissue edema. Degeneration, disorganization of germinal epithelial cells, and loss of spermatogenesis lead to an opposite effect on the Leydig cells responsible for the secretion of the hormone testosterone, which affects the hypothalamus–pituitary axis [33,34].

In the current study, rats treated with sodium fluoride had significantly lower androgen receptor expression levels in their testicles. In a related study [35], it was found that *Artemisia absinthium* has the ability to reduce the toxicity of NaF and that it leads to minor but significant immunostaining for androgen in the Leydig and peritubular cells in albino rat testes. The extract also helps prevent oxidative stress and the formation of lipid peroxides by scavenging free radicals, which are toxic byproducts of multiple metabolic activities in biological cellular membranes [36]. Thus, the biological activities of phenolic compounds and flavonoids are associated with their antioxidant potential, selectively acting on the developing germinal epithelial cells. This leads to the amelioration of AR immunostaining occurring with a new generation of Leydig cells, as shown by the presence of immunoreactive 3B-HSD, with a progressive increase in testosterone production during long-term recovery [13,37,38]. This study’s results suggest that fluoride exposure causes severe testis tissue damage. *A. absinthium* is an essential candidate for the preservation of testicular function after NaF exposure; this may be due to amelioration of the hormone levels and androgen receptor expression in the testis.

Investigating several facets of *A. absinthium*’s efficacy is necessary to investigate how well its ethanolic extract protects rat testes against sodium fluoride toxicity. Although this research may yield encouraging findings, it is important to take into account several limitations. The content of plant extracts can change depending on a number of variables, including the time of harvest, the location, and the extraction technique. Results from various studies may differ due to the difference in phytochemicals (such as flavonoids, terpenoids, and essential oils) found in *A. absinthium*. The mechanism by which the ethanolic extract of *A. absinthium* mitigates sodium fluoride toxicity is not always fully understood. Although some of the plant’s active chemicals, such as antioxidants, may have protective effects, further research is necessary to fully understand the molecular mechanisms behind their potential for therapeutic use. Finally, although *A. absinthium* may have potential effects in reducing sodium fluoride toxicity, the aforementioned limitations underscore the necessity of additional research to confirm its effectiveness, safety, and suitability for health.

## 5. Conclusions

The administration of *A. absinthium* extract with sodium fluoride led to reduced testis weights and lipid peroxidation levels and increased serum LH, FSH, and testosterone concentrations. The expression of androgen receptors in the treated rats’ testes also significantly increased, and testicular histological changes were ameliorated in rats treated with the extract. Therefore, treatment with *Artemisia absinthium* extract has positive effects on male reproductive function by inhibiting fluoride toxicity, maybe via ameliorative testicular function.

## Figures and Tables

**Table 1:** Effects of ethanolic extract of *A. absinthium* on body and testis weights under NaF toxicity in rats (mean ± SD).

Group	Body Weight (mg)	Weight of Testis (mg)
I	188.50 ^a^ ± 2.428	1.7067 ^a^ ± 0.018
II	192.83 ^a^ ± 3.86	1.7150 ^a^ ± 0.030
III	160.17 ^c^ ± 4.445	0.8983 ^c^ ± 0.0222
IV	171.40 ^b^ ± 3.209	1.4820 ^b^ ± 0.040

Different letters indicate differences among groups at *p* ≤ 0.05.

**Table 2:** Effect of ethanolic extract of *A. absinthium* on levels of follicle-stimulating hormone (FSH), luteinizing hormone (LH), testosterone, and malonedialdehyde (MDA) under NaF toxicity in rats.

Group	FSH (mlU/mL)	LH (mlU/mL)	Testosterone ng/mL	MDA (nmol/mg of pt)
I	1.388 ^a^ ± 0.0116	1.186 ^a^ ± 0.0103	1.678 ^a^ ± 0.0116	4.096 ^c^ ± 0.0355
II	1.366 ^a^ ± 0.0103	1.163 ^a^ ± 0.0102	1.646 ^a^ ± 0.0150	4.130 ^c^ ± 0.0275
III	0.798 ^c^ ± 0.0160	0.381 ^c^ ± 0.0365	0.605 ^c^ ± 0.0187	7.160 ^a^ ± 0.0726
IV	1.074 ^b^ ± 0.0288	0.864 ^b^ ± 0.027	1.138 ^b^ ± 0.0130	5.506 ^b^ ± 0.0841

Different letters indicate differences among groups at *p* ≤ 0.05.

**Table 3:** Effect of ethanolic extract of *A. absinthium* on seminiferous tubule diameters and germinal epithelial heights under NaF toxicity in rats.

Group	Diameter of Seminiferous Tubules (μm)	Germinal Epithelial Height (μm)
I	169.85 ^b^ ± 1.545	55.04 ^b^ ± 0.857
II	173.58 ^a^ ± 1.191	59.41 ^a^ ± 1.04
III	157.37 ^d^ ± 1.950	45.39 ^c^ ± 0.937
IV	165.766 ^c^ ± 1.355	52.65 ^b^ ± 0.936

Different letters indicate differences among groups at *p* ≤ 0.05.

## Data Availability

All data generated or analyzed during this study are included in this manuscript.
